# Natural Terpenes Prevent Mitochondrial Dysfunction, Oxidative Stress and Release of Apoptotic Proteins during Nimesulide-Hepatotoxicity in Rats

**DOI:** 10.1371/journal.pone.0034200

**Published:** 2012-04-03

**Authors:** Brijesh Kumar Singh, Madhulika Tripathi, Bhushan P. Chaudhari, Pramod K. Pandey, Poonam Kakkar

**Affiliations:** 1 Herbal Research Section, CSIR-Indian Institute of Toxicology Research (Formerly-Industrial Toxicology Research Centre), Lucknow, Uttar Pradesh, India; 2 Pathology Laboratory, CSIR-Indian Institute of Toxicology Research (Formerly-Industrial Toxicology Research Centre), Lucknow, Uttar Pradesh, India; 3 Department of Biotechnology, Bundelkhand University, Jhansi, Uttar Pradesh, India; Medical University of South Carolina, United States of America

## Abstract

Nimesulide, an anti-inflammatory and analgesic drug, is reported to cause severe hepatotoxicity. In this study, molecular mechanisms involved in deranged oxidant-antioxidant homeostasis and mitochondrial dysfunction during nimesulide-induced hepatotoxicity and its attenuation by plant derived terpenes, camphene and geraniol has been explored in male Sprague-Dawley rats. Hepatotoxicity due to nimesulide (80 mg/kg BW) was evident from elevated SGPT, SGOT, bilirubin and histo-pathological changes. Antioxidants and key redox enzymes (iNOS, *mt*NOS, Cu/Zn-SOD, Mn-SOD, GPx and GR) were altered significantly as assessed by their mRNA expression, Immunoblot analysis and enzyme activities. Redox imbalance along with oxidative stress was evident from decreased NAD(P)H and GSH (56% and 74% respectively; *P*<0.001), increased superoxide and secondary ROS/RNS generation along with oxidative damage to cellular macromolecules. Nimesulide reduced mitochondrial activity, depolarized mitochondria and caused membrane permeability transition (MPT) followed by release of apoptotic proteins (AIF; apoptosis inducing factor, EndoG; endonuclease G, and Cyto *c*; cytochrome c). It also significantly activated caspase-9 and caspase-3 and increased oxidative DNA damage (level of 8-Oxoguanine glycosylase; *P*<0.05). A combination of camphene and geraniol (CG; 1∶1), when pre-administered in rats (10 mg/kg BW), accorded protection against nimesulide hepatotoxicity *in vivo,* as evident from normalized serum biomarkers and histopathology. mRNA expression and activity of key antioxidant and redox enzymes along with oxidative stress were also normalized due to CG pre-treatment. Downstream effects like decreased mitochondrial swelling, inhibition in release of apoptotic proteins, prevention of mitochondrial depolarization along with reduction in oxidized NAD(P)H and increased mitochondrial electron flow further supported protective action of selected terpenes against nimesulide toxicity. Therefore CG, a combination of natural terpenes prevented nimesulide induced cellular damage and ensuing hepatotoxicity.

## Introduction

Mitochondria play a central role in regulating cell survival and death signaling in liver cells. Rising evidences suggest that apoptotic pathways converge at the mitochondria, where signaling is initiated through a series of molecular events culminating in the release of death factors [1]. This triggers either caspase-dependent or independent apoptosis. Mitochondrial apoptotic proteins like cytochrome *c* (Cyt *c*), cause caspase-dependent cell death and trigger caspase-9 activation by binding and activating apoptotic protease activating factor-1 (ApoAF-1). This event potentiates caspase activation by binding inhibitor of apoptosis proteins (IAP) and blocking their caspase-inhibitory activity. Apoptosis-inducing factor (AIF) and endonuclease G (EndoG), along with other important mitochondrial proapoptotic proteins, are reported to translocate to the nucleus and cause oligonucleosomal DNA fragmentation during mitochondria mediated caspase-independent cell death [1], [2]. Release of these mitochondrial death effectors is tightly regulated by the Bcl-2 family proteins during membrane permeability transition (MPT). It is clearly a redox sensitive process in which membrane protein thiols (Cys-56 of adenine nucleotide translocator; ANT) get oxidized and cross-linked; leading to increased MPT pore opening [3], [4]. This phenomenon is also sensitive to glutathione depletion in a synchronized manner with oxidative stress and calcium overload [3], [5]. Furthermore, threshold mitochondrial damage with oxidative stress causes oxidative DNA damage and increased 8-oxoguanine-DNA glycosylase (OGG; DNA repair enzyme) to overcome cellular damage [6].

Many diseases and metabolic disorders including Alzheimer’s, Parkinson’s, liver disorders are caused by oxidative stress and mitochondrial dysfunction, providing evidence that mitochondrial impairment can be deleterious [4], [7]. Exposure of nonsteroidal anti-inflammatory drugs (NSAIDs) is reported to have inhibitory effects on mitochondrial respiration and uncoupling [7], [8]. Severity of mitochondrial impairment is now believed to have a direct co-relation with the nature of NSAIDs which is being further explored [9], [10].

**Figure 1:**
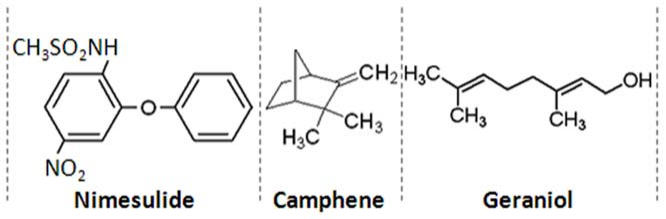
Structure of the drug - nimesulide, and camphene and geraniol. Nimesulide is a non-steroidal anti-inflammatory drug (NSAID). Camphene is a bicyclic mono-terpenoid whereas geraniol is acyclic monoterpene-alcohol.

Nimesulide (N-(4-Nitro-2-phenoxyphenyl) methanesulfonamide; Figure 1-Nimesulide) is a selective cyclooxygenase-2 (COX-2) inhibitor and has better gastro-intestinal tolerability among other NSAIDs in its class [11]. The drug is prescribed for the management of symptomatic pain in conditions like osteoarthritis and primary dysmenorrhoea in patients above 12 years of age, but cases of severe hepatotoxicity have also been reported [12]. Therefore, it is rational to explore the molecular mechanisms involved in nimesulide-induced mitochondrial impairment resulting in liver toxicity, and at the same time, search for a supplement preferably of natural origin, that could overcome the side effect of this otherwise effective drug.

Natural/herbal formulations which include phytochemicals are traditionally used in the treatment of liver disorders. Studies carried out in our laboratory revealed significant protection against acetaminophen induced hepatotoxicity by probiotics [13]. Standardized extract of *Fumaria parviflora* Lam. was also found to reduce drug-induced hepatotoxicity by intervening the critical events in apoptosis [14], [15]. Glycyrrhizic acid, an active constituent of *Glycyrrhiza glabra* Linn. was also found to modulate t-BHP induced apoptosis in rat primary hepatocytes [16]. Plant derived metabolites such as alkaloids, terpenes, flavanoids, isoflavones, saponins etc. have beneficial effects on health in the treatment of conditions such as asthma, eczema, rheumatoid arthritis, chronic fatigue [17], [18]. Silymarin, a flavonoid complex extracted from the seeds of *Silybum marianum* (commonly known as ‘milk thistle’; family - Asteraceae), is recognized as a hepato-protective agent of herbal origin in Europe, USA and other countries. It also has clinical applications in the treatment of toxic hepatitis, fatty liver, cirrhosis, ischemic injury, and viral hepatitis via its anti-oxidative, anti-lipid peroxidative, antifibrotic, anti-inflammatory and liver regenerating effects [13], [14], [16]. In our study, silymarin was used as positive control for hepato-protection. Phyto-constituents used in this study i.e. camphene (Figure 1-Camphene) possesses antilithic and expectorant properties and geraniol (Figure 1-Geraniol) is reported to prevent cancer and have antimicrobial as well as antioxidant activities [19]. The aim of the present study was to explore protective role of a combination of terpenes, camphene and geraniol (CG), in nimesulide-induced deranged mitochondrial function, responsible for cell death during hepatotoxicity.

## Materials and Methods

### Chemicals

All chemicals used in the study were obtained from Sigma Chemicals Co. (St. Louis, MO, USA) unless indicated otherwise. The reagents were prepared in de-ionized ultra-pure water (Direct Q5, Millipore, Banglore, India).

### Animals

Male Sprague-Dawley rats weighing 180±20 g were taken from Indian Institute of Toxicology Research (IITR) animal colony and used for the experiments. Animals were fed standard commercial pellet diet and water *ad libitum.* Rats were kept under standard conditions of humidity (60–70%), temperature (25±2^°^C) and a controlled 12 h light/dark cycle. All the guidelines of Institutional Animal Ethics Committee (ITRC/IAEC/15/2008) were followed while handling the animals.

### Study Plan and *in vivo* Treatment Schedule

*In vivo* study was divided in two parts: A- was used to analyze the histopathology, serum clinical biochemistry and molecular parameters, whereas, B- was conducted to isolate hepatic mitochondria and conduct related experiments.

Rats were randomized and divided into six groups. Nimesulide (100 mg/ml) was dissolved in dimethyl sulfoxide (DMSO) and filtered (0.22 µm). Concentration of DMSO used was nontoxic and has been used earlier for oral administration of water-insoluble compounds [15]. DMSO (GROUP-I; 1 µl/g BW/day) and nimesulide (80 mg/kg BW/day) was administered *po* once daily for 5 days. The nimesulide dose used here in rats is 2.5 times lower than the oral LD_50_ dose for rats (200 mg/kg BW) [20]. 80 mg/kg BW/day dose was selected based on our pilot studies which showed that nimesulide at higher dose (100 mg/kg BW) was lethal in rats while 50 mg/kg did not show significant toxicity. 80 mg/kg BW dose showed significant hepato-toxicity without any mortality, which could be monitored. The selected nimesulide dose in this study is also 3.9 times higher than human equivalent dose when corrected for interspecies differences with the dose scaling factor of 6.167 [21]. This was done to create a condition of overdosage as upto 4 times therapeutic dose is reported to be well tolerated with minor renal toxicity [22]. Camphene and geraniol, in combination (CG; 1∶1) (5+5 = 10 mg/kg), and silymarin (Sil) (100 mg/kg) were dissolved in 10% DMSO and administered *po* once daily for 5 days. The pre-treatment regime of this study was based on our previous *in vivo* [15] and *in vitro* studies [14] which showed best protection during pre-treatment of plant extract or phytochemicals in nimesulide stressed rats and primary rat hepatocytes. Our *in silico* investigations revealed that there were some common interactive targets for ‘C’, ‘G’ and nimesulide involving cytosolic and mitochondrial proteins (unpublished data). To overcome any possibility of competitive inhibition or drug - phytochemical interaction, co-treatment was avoided. It was also observed that combination of camphene and geraniol rendered significantly higher cyto-protection than treatment with individual terpenes and response was observed with lesser concentration of CG (unpublished data). *In vivo* dosage of CG combination is based on pilot study and selection of effective but non-toxic dose was made which incidentally was 1/10^th^ of silymarin dose. Details of dosage schedule and division of treated groups of rats is given in table 1.

**Table 1:** Treatment schedule and division of groups.

Groups	Treatment and Dosage
**Group I**	Control rats treated with vehicle (DMSO; 1 µl/g BW/day)
**Group II**	Nimesulide (80 mg/kg BW/day)
**Group III**	CG combination (5+5 mg ( = 10 mg)/kg BW/day)
**Group IV**	Silymarin (100 mg/kg BW/day)
Treatment was given for 5 days (once daily).	
**Group V**	Pre-treatment with CG combination for 5 days followed by nimesulide (80 mg/kg BW/day) for five days (once daily)
**Group VI**	Pre-treatment with silymarin for 5 days followed by nimesulide (80 mg/kg BW/day) for five days (once daily)
Animals of all the groups were sacrificed on the same day.	

The overnight fasted rats were euthanized by cervical dislocation. Blood was collected by cardiac puncture, and serum separated as per standard protocol. Liver was quickly excised; one portion was used for histopathology, 2–3 g of tissue was used for isolation of mitochondria, 1 g tissue was preserved in RNA stabilization solution (Qiagene, GmbH, Germany) for RNA isolation and remaining tissue was snap frozen in liquid nitrogen for biochemical and molecular analysis.

### Evaluation of Serum Biomarkers and Histopathology of Liver

Liver transaminases (SGOT, SGPT) and bilirubin in serum were analyzed on automated biochemical analyzer (Chemwell Awareness Technologies, Inc., Germany) with kits purchased from Spinreact (Spain).

Approximately, 1 g of liver tissue section was randomly cut and fixed in 10% neutral buffered formalin. The tissue was embedded in paraffin block and cut in 5 µm sections using motorized rotary microtome (RM 2155, Leica, Germany). After staining with haematoxylin and eosin (H&E), slides were examined under compound light microscope and photographed using Leica DCF-280 camera controlled with software (Leica application suite Ver.3.5.0, Leica, Germany) to assess histopathological changes.

### Isolation of Liver Mitochondria and Preparation of Sub-cellular Fractions

Standardized protocol for isolation of mitochondria was used as described earlier [5] with some modifications. In brief, 2–3 g liver tissue was homogenized (10% W/V) in ice-cold MSH buffer (10 mM HEPES, pH-7.5, containing 200 mM mannitol, 70 mM sucrose, 1.0 mM EGTA and 2.0 mg/ml fatty acid free serum albumin). Resulting homogenate was centrifuged (Sigma-3K18, Germany) at 500 g for 10 min at 4°C. Supernatant was collected and again centrifuged at 15,000 g for 10 min at 4°C to obtain crude mitochondrial pellet. Supernatant was recovered (for cytosolic fraction) and the pellet was washed three times with ice-cold homogenization buffer to get intact mitochondria taking into account the findings of Kruglov et al. [23]. Isolated mitochondria were then sonicated (3 cycles of 20 s each with 5 s time interval) (Labsonic® M, Sartorius, Germany) and centrifuged at 15,000 g for 10 min at 4°C to obtain mitochondrial fraction. Recovered supernatant (for cytosolic fraction) was also centrifuged at 22,000 g for 15 min at 4°C to obtain cytosolic fraction. Mitochondrial and cytosolic fractions were checked for purity by immunoblotting using voltage dependent anion channel (VDAC) as mitochondrial marker and β-actin as cytosolic marker. No contamination of either marker was observed in respective fractions. Loose mitochondrial fraction was discarded.

### Treatments in Isolated Mitochondria

Isolated mitochondria were treated with *tert*-butyl hydroperoxide (t-BHP), N-ethylmaleimide (NEM) and CaCl_2_. NEM, an alkylating agent (1 mM) was used to deplete thiol whereas t-BHP (250 µM) and CaCl_2_ (100 µM) were used to produce oxidative stress and Ca^2+^ overload conditions respectively [5], [24]. KH_2_PO_4_ (*Pi*: 2 mM) was used as positive control for mitochondrial membrane permeability transition (MPT) and CyclosporinA (CsA; 1 µM) as MPT blocker [25].

#### Preparation of mitochondrial suspension and protein estimation

Intact mitochondrial pellet was suspended in suspension buffer (5 mM 3-(N-morpholino) propanesulfonic acid (MOPES), containing 75 mM sucrose, 225 mM mannitol and 1 mM ethylenediaminetetraacetic acid (EDTA), pH-7.4) for mitochondrial electron flow and in respiration buffer (210 mM mannitol, 70 mM sucrose, 5 mM 4-(2-hydroxyethyl)-1-piperazineethanesulfonic acid (HEPES), 7.5 mM succinate, pH-7.4 with 1M KOH) for mitochondrial swelling, flow cytometric analysis and other biochemical experiments yielding a suspension of 30–40 mg mitochondrial protein/ml. Protein estimation was done according to the method of Lowry et al. [26]. 150 µg protein/ml was used to observe mitochondrial swelling and 40 mg mitochondrial protein/ml was used for other enzymatic assays and flow cytometric analysis.

### Quantification of Antioxidant Enzymes Activity

Superoxide dismutase (SOD) activity was determined using the method of Kakkar et al. [27]. 50% inhibition of formazan formation in 1 min was considered as one unit of activity. Glutathione peroxidase (GPx) and Glutathione reductase activity were assayed as described earlier [15]. Absorbance was measured on Ultrospec 3100*pro* UV/Visible spectrophotometer (Amersham Biosciences, Sweden) at 25°C and 1 µM NADPH oxidized/min/mg protein was considered as 1 U activity of GPx and GR.

### Determination of Antioxidant Gene Expression

mRNA expression of antioxidant enzymes was assessed by reverse transcriptase-polymerase chain reaction (RT-PCR) [28]. Total RNA was extracted from the liver using TRIzol® reagent (Invitrogen, Carsland, CA, USA) according to the manufacturer’s protocol and treated with DNase I for 15 min at 37°C. First strand cDNA was synthesized using SuperScript® III First-Strand synthesis system (Invitrogen, Carsland, CA, USA). Synthesized cDNA was subsequently used for further amplification using suitable primers (as in Table 2). PCR products were electrophoresed on 2.0% agarose gel, visualized and image analyzed on gel documentation system (Alfa Innotech equipped with Alpha Imager software, CA, USA). Densitometry of the bands was performed on ImageJ software (1.41o, NIH, USA). β-actin gene was used as internal control.

**Table 2:** List of primers, annealing temperature and product size.

Gene	Forward (F)/Reverse (R) primer	Annealing temperature	Product size	Reference
iNOS	F-CATTGAGATCCCGAAACGCTAC	61°C	∼140 bp	[39]
iNOS	R-AGCCTCATGGTGAACAGTTCT	61°C	∼140 bp	[39]
Cu/Zn-SOD	F-CGTCATTCACTTCGAGCAGA	55°C	∼341 bp	[28]
Cu/Zn-SOD	R-CACCTTTGCCCAAGTCATCT	55°C	∼341 bp	[28]
Mn-SOD	F-CGTGCTCCCACACATCAATC	66°C	∼380 bp	[40]
Mn-SOD	R-TGAACGTCACCGAGGAGAAG	66°C	∼380 bp	[40]
GPx	F-CAGTTCGGACATCAGGAGAAT	60°C	∼139 bp	[41]
GPx	R-AGAGCGGGTGAGCCTTCT	60°C	∼139 bp	[41]
GR	F-GGGCAAAGAAGATTCCAGGTT	60°C	∼101 bp	[41]
GR	R-GGACGGCTTCATCTTCAGTGA	60°C	∼101 bp	[41]
β-Actin	F-TGAGAGGGAAATCGTGCGT	61°C	∼225 bp	[39]
β-Actin	R-CATTGAGATCCCGAAACG	61°C	∼225 bp	[39]

### Flow Cytometric Analysis

Flow cytometer (BD-LSR; Becton Dickinson and Company) was used to measure superoxide (O_2_ **^·-^**), secondary ROS/RNS (HO**^·-^/**OONO^-^), thiol, and change in mitochondrial membrane potential (?Ψm) or mitochondrial depolarization (Ψm_Low_) using corresponding fluorescent probes described below and data analyzed using Cell Quest software supplied with the instrument. Signals were obtained using corresponding band pass filters. Each determination is based on mean fluorescence intensity of 10,000 events.

#### Superoxide and secondary ROS/RNS determination in mitochondria

Level of superoxide and secondary ROS/RNS inside mitochondria were measured as fluorescence of ethidium (ETH) and dichlorofluorescein (DCF) respectively as described earlier [5]. In brief, mitochondria from control and treated groups were incubated for 30 min with DHE (5 µM) and DCFH-DA (10 µM) at 37°C in dark. Signals were obtained using a 585-nm band pass filter (FL-2 channel) for ETH and a 530-nm band pass filter (FL-1 channel) for DCF.

#### Mitochondrial thiol determination

CellTracker^™^ Green CMFDA (Molecular Probes, Eugene, Oregon, USA) was used to analyze thiol content [4]. In mitochondria, glutathione level is high (5–10 mM) which represents to major thiol pool [2]. Mitochondria/primary hepatocytes (isolated using two step collagenase perfusion method as described earlier by Tripathi et al. [14], [15]) were incubated for 30 min with the CellTracker^™^ Green CMFDA (1 µM) at 37°C in dark. The reagent is transformed into a cell-impermeant fluorescent adduct that can be detected on flow cytometer. Signals were obtained using 530 nm band pass filter (FL-1 channel).

#### Changes in mitochondrial membrane potential (ΔΨm)

ΔΨm was assessed with JC-1 dye on flow cytometer as described earlier [14]. Mitochondrial samples were incubated in dark for 30 min at room temperature with 5 µM dye. Signals were obtained using FL-1 and FL-2 channel settings.

### Protein Carbonyl Quantification

Protein carbonyl, marker of oxidative damage of proteins, was analyzed spectrophotometrically at 360 nm by the method of Levin et al. [29] with some modifications. Final pellet of protein carbonyl from samples and blanks was dissolved in 500 µl of Guanidine HCl (6 M), centrifuged at 10,000 g for 10 min at 4^°^C, 200 µl of supernatant was taken out in a 96 well plate and absorbance was read at 360 nm using UV/visible spectrophotometer at 25°C. Bovine serum albumin (BSA) was used as standard.

### Determination of Mitochondrial Membrane Lipid Peroxidation (Quantification of MDA)

Membrane lipid peroxidation (malondialdehyde, MDA formation) was done as described earlier [15]. The pink colored MDA-thiobarbituric acid (TBA) adduct was read at 532 nm and all nonspecific adducts at 600 nm. Lipid peroxidation was expressed as nmol of MDA formed/mg protein of mitochondria using a standard curve of 1,1,3,3-tetraethoxypropane.

### Determination of Reduced Mitochondrial Pyridine Nucleotide (NAD[P]H) Levels

Level of mitochondrial NADH and NADPH (represented as NAD(P)H and taken as mitochondrial function), was assessed as UV excited blue auto-fluorescence on spectrofluorometer (Varioskan Flash 4.00.53 supplied with SkanIt Software 2.4.3.37 Research Edition; Thermo Fisher Scientific, Finland) using Ex/Em: 380/450 nm [4].

### Determination of Mitochondrial Electron Flow (3-(4,5-Dimethylthiazol-2-yl)-2,5-diphenyltetrazolium Bromide; MTT Reduction Assay)

Experiment was carried out according to Cohen and Kesler [30] with some modifications. The samples were quenched with DMSO and the absorbance was measured at 592 nm. ELISA plate reader (Synergy HT, Biotek, USA) was used to read mitochondrial samples in a 48-well plate.

### Immunoblot Analysis

Proteins (40 µg) were separated on SDS-PAGE and electro-blotted on polyvinylidene fluoride (PVDF) membrane as described earlier [14]. Anti-Cyt *c*, AIF, EndoG, Mn-SOD, mitochondrial nitric oxide synthase (*mt*NOS: an analogue of neuronal NOS), caspase-9, caspase-3, 8-oxoG DNA glycosylase (OGG1/2; Santa Cruz Biotechnology, Inc) and inducible NOS (iNOS) primary antibodies (1°Ab) were used in 1∶500 dilutions whereas Anti-VDAC, β-actin, iNOS, nitrotyrosine 1°Ab and horse radish peroxidase-conjugated secondary antibody (2°Ab) (Santa Cruz Biotechnology, Inc) were used in 1∶1000 dilutions. Anti-cytochrome *c* oxidase subunit-IV (Cyto-Ox-IV) 1°Ab (Abcam, Cambridge, UK) was used in 1∶2000 dilution. Quantification of bands was done using ImageJ software (version 1.41o, NIH, USA). Values are normalized with internal loading control of corresponding fractions (i.e. β-actin for cytosolic and Cyto-Ox-IV for mitochondria).

### Determination of Mitochondrial Swelling

Mitochondrial swelling was determined in 96 well plate according to Tay et al. [31] with some modifications. Mitochondrial pellet was suspended in respiration buffer. 150 µg mitochondrial protein was used in 300 µl assay system. The swelling was recorded as decrease in the absorbance at 1 min interval for a period of 20 min at 540 nm using microplate based UV/visible spectrophotometer (SpectramaxPlus 384, Molecular Devices, USA). Percent swelling was calculated with respect to *Pi* (2 mM KH_2_PO_4_) induced swelling (*Pi* = 100% swelling) and CsA (1 µM) was used as MPT blocker.

### Statistical Analysis of Data

The data are reported as mean ± standard deviation (SD) (n = 6). The probability values (*P*) were derived using Analysis of variance (ANOVA) followed by Tukey test (SPSS 14.0 for Windows, USA), wherein the differences were considered to be significant at *, # *P*<0.05; **, ## *P*<0.01; and ***, ### *P*<0.001 (*-compared with control, #-compared with stress).

## Results

### Administration of Nimesulide caused Hepatotoxicity in SD Rats

**Figure 2:**
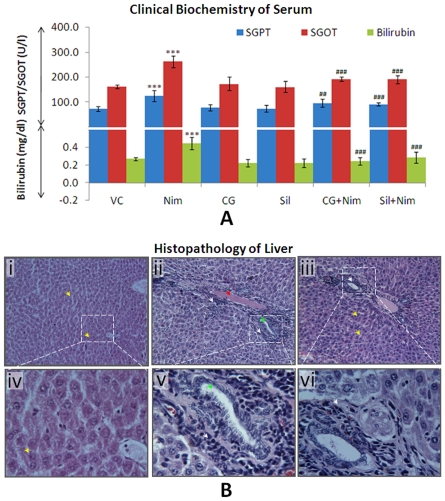
Determination of nimesulide-induced hepatotoxicity. A. Clinical biochemistry (levels of SGPT, SGOT and bilirubin) of blood serum. **B.** Histopathology of H&E stained liver tissue of vehicle control (**i**), nimesulide administered rats (**ii**) and CG pre-administered rats (**iii**). Pictures were taken at 125X (i, ii, and iii) and 500X (iv, v and vi) magnification. In figures yellow arrow heads represent normal hepatocytes; red arrowhead represents edema, green arrowhead represents hyperplastic bile ductule, and white arrowheads represent degenerating hepatocytes with infiltration of inflammatory cells. Level of significance is denoted as */# *P*<0.05, **/## *P*<0.01 and ***/### *P*<0.001. *, compared to vehicle control and #, compared to nimesulide stress.

Nimesulide treated group showed significant (*P<0.001) increase in SGPT (68%), SGOT (62%) along with serum bilirubin levels (66%) when compared to vehicle control (Figure 2A), indicating nimesulide-induced hepatotoxicity. In CG and silymarin administered groups SGPT, SGOT and bilirubin showed non-significant changes. Pre-administration of CG significantly prevented nimesulide-induced alterations in these biochemical parameters i.e. SGPT (30%; ^#^ *P*<0.01) and SGOT (19%; ^#^ *P*<0.001). Similar response was observed in silymarin pre-treated group. Bilirubin content was also comparable to vehicle control (^#^ *P*<0.001) in CG pre-treated animals.

Figure 2B shows histo-pathological changes in liver tissues of nimesulide stressed and CG pre-administered nimesulide treated groups. Figure 2B-i and 2B-iv revealed no pathological changes in vehicle control group i.e. normal hepatic cells with well-preserved cytoplasm and prominent nucleus, were observed. The histology of nimesulide administered animals (Figure 2B-ii and 2B-v) showed degenerating hapatocytes infiltrated with inflammatory cells, hyperplastic bile ductules and edema. Liver section of group which was pre-administered with CG (Figure 2B-iii and 2B-vi) showed comparatively normal hepatocytes while infiltration of mixed inflammatory cells was also minimum.

The serum biochemical parameters and histopathology of liver revealed that nimesulide-induced hepatotoxicity could be prevented using a combination of camphene and geraniol. To explore involvement of oxidant-antioxidant homeostasis and mitochondrial function, further experiments were done in isolated mitochondria from different treatment groups.

### Alterations in Oxidant-antioxidant Homeostasis

#### CG prevented nimesulide-induced alterations in antioxidant defense at transcriptional level

Nimesulide was found to significantly hamper the antioxidant defense machinery (Table 3). It caused 74% (*P<0.001) thiol depletion in mitochondria of treated rats (compared to 89% in hepatocytes; data not shown). SOD, GPx and GR activities in mitochondria were found to be decreased by 58% (*P<0.001), 48% (*P<0.01) and 23% (*P<0.05) respectively when compared to control whereas 32%, 36% and 33% (*P<0.01) decrease was observed in cytosol. CG pre-administration significantly prevented this reduction in antioxidant activities. Only 14% decrease in *mt*SOD activity and 20% in Cu/Zn-SOD was observed. Redox regulatory enzymes, GPx (15%) and GR (6%) activities also decreased marginally in mitochondria. In silymarin pre-administered rats, this decrease was 20% *mt*SOD, 18% GPx and 13% GR activity, respectively. Disturbance in thiol level was also comparatively low in mitochondria of CG and silymarin pre-administered rats (38% and 28% depletion, ^#^ *P*<0.01).

**Table 3:** GSH content and Antioxidant enzyme activities.

Treatments	GSH (MFI of CMF)	SOD (U/min/mg protein)		GPx (U/min/mg protein)		GR (U/min/mg protein)	
Fractions	Mitochondria	Mitochondria	Cytosol	Mitochondria	Cytosol	Mitochondria	Cytosol
VC	369±23	6.9±0.4	55.0±4.2	0.93±0.06	10.0±0.8	0.63±0.01	9.4±0.5
Nim	97±8^***^	2.9±0.4^***^	37.3±2.1^**^	0.49±0.07^**^	6.3±0.5^**^	0.49±0.02^*^	6.3±0.3^**^
CG	360±19	6.3±0.7	53.6±3.8	0.93±0.05	9.6±0.7	0.63±0.02	9.2±0.4
SIL	370±21	7.1±0.7	51.0±3.3	0.91±0.03	9.0±0.6	0.61±0.01	9.1±0.5
CG+Nim	238±28^##^	5.9±0.4^##^	44.0±2.3^##^	0.79±0.08^##^	8.0±0.6^#^	0.59±0.03^##^	8.0±0.4
Sil+Nim	269±23^##^	6.0±0.3^##^	44.1±2.4^##^	0.84±0.03^##^	8.1±0.7^#^	0.54±0.02^##^	8.1±0.4^#^

Mitochondrial thiol (majorly GSH) was assessed using CellTracker^™^ Green CMFDA fluorescent probe on flow cytometer. Data represent mean fluorescent intensity (MFI) of chloromethylfluorescein (CMF). Superoxide dismutase (SOD), glutathione peroxidase (GPx) and glutathione reductase activity of mitochondrial and cytosolic fractions were demonstrated as unit activity/min/mg protein. Level of significance is denoted as */# *P*<0.05, **/## *P*<0.01 and ***/### *P*<0.001. *, compared to vehicle control and #, compared to nimesulide stress.

**Figure 3:**
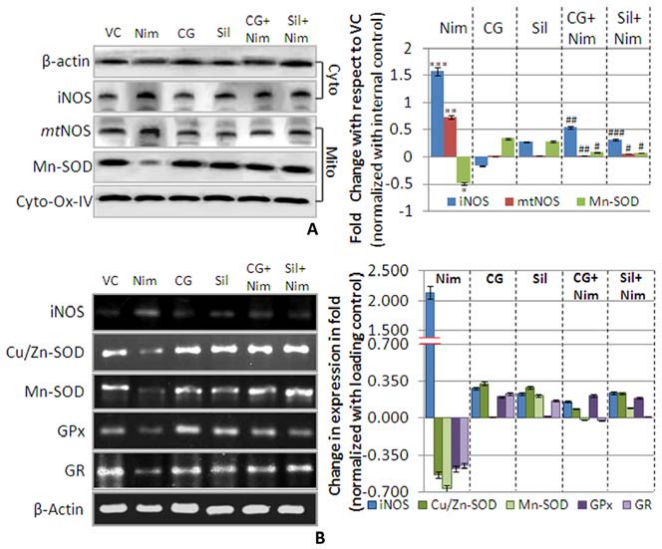
Antioxidant defense. A. Immunoblot analysis of key antioxidant enzyme in mitochondria, Mn-SOD including *mt*NOS (an analogue of nNOS) in mitochondrial fraction whereas iNOS in cytosolic fraction. β-Actin was used as internal loading control for cytosolic fraction whereas Cyto-Ox-IV for mitochondrial fraction. All the densitometric values are normalized with respective internal loading control. Densitometry of bands was done using ImageJ software (V1.41o, NIH, USA). Bar graph in right panel represents protein levels. Values are represented as compared to vehicle control in folds change. **B.** mRNA expression of antioxidant enzymes superoxide dismutases (Cu/Zn-SOD and Mn-SOD), glutathione peroxidase (GPx) and glutathione reductase including nitric oxide synthase inducible (iNOS) were assessed by RT-PCR. β-Actin and Cyto-Ox-I were used as internal loading control. All the densitometric values are normalized with respective internal loading control. Bar graph in left panel represents mRNA levels. Values are represented as compared to vehicle control in folds change. Level of significance is denoted as */# *P*<0.05, **/## *P*<0.01 and ***/### *P*<0.001. *, compared to vehicle control and #, compared to nimesulide stress.

NOS, an important enzyme for regulating nitrosative stress, was evaluated along with other antioxidant enzymes like SOD, GPx and GR to assess ROS/RNS induced stress. Immunoblot analysis (Figure 3A), revealed significant increase in iNOS (1.50 fold, *P<0.001) and *mt*NOS (0.73 fold, *P<0.01) levels whereas Mn-SOD was significantly decreased (49%,*P<0.05). CG pre-administration prevented nimesulide-induced alterations in oxidative stress regulatory enzymes, not only at transcriptional but at translational level also. Levels of iNOS and *mt*NOS were decreased by 96% and 71% respectively (^#^ *P*<0.001 and *P*<0.01) showing a significant protection. Pre-administration of silymarin, a known hepato-protectant, also reduced nimesulide-induced alterations. iNOS (^#^ *P*<0.001) and *mt*NOS (^#^ *P*<0.05) decreased significantly while Mn-SOD increased by 55% (^#^ *P*<0.05).

Transcription level changes (mRNA expression) in these antioxidant genes (iNOS, Cu/Zn-SOD, Mn-SOD, GPx and GR) were evaluated to assess the toxic response (Figure 3B). In nimesulide administered rats iNOS gene expression increased by 2.13 fold whereas the expression of Cu/Zn-SOD and Mn-SOD, decreased by 53% and 66% respectively when compared to control. A decrease in GPx (48%) and GR (45%) mRNA expression was also observed indicating perturbed antioxidant defense.

In CG and silymarin pre-administered rats, a respective 2.0 (i.e. 200%) and 1.9 fold (i.e. 190%) decrease in iNOS expression was observed when compared to nimesulide treated group. CG and silymarin pre-administration increased mRNA expression of Cu/Zn-SOD and Mn-SOD (62% and 64% respectively), which was comparable to response with silymarin (76% and 75% respectively). Increased levels of GPx and GR in CG and Silymarin pre-administered groups were also observed (69% and 43% for CG; 67% and 47% for Sil) when compared to nimesulide treated group.

#### CG overwhelmed nimesulide-induced oxidative stress and related damage to mitochondrial lipids and proteins

**Figure 4:**
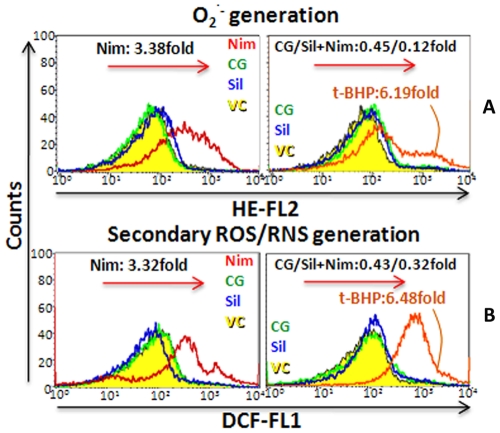
Oxidative stress status. A. superoxide (O_2_^·-^) and, **B**. secondary ROS/RNS generation were assessed using DHE and DCFH-DA dye on flow cytometer. t-BHP (oxidative stress generator) was used as positive control for oxidative stress in mitochondria.

Flow cytometric analysis (Figure 4A and B) revealed that nimesulide caused significant increase (3.38 and 3.32 fold, *P<0.001, as compared to control) in superoxide and secondary ROS/RNS generation in mitochondria of treated tissue. CG and silymarin when administered to unstressed rats did not cause any significant change. CG and silymarin pre-administration significantly (^#^ *P*<0.001) prevented superoxide and ROS/RNS generation during nimesulide stress and it was found to enhance only 0.45 and 0.43 fold in CG treated group while 0.12 and 0.32 fold for silymarin respectively. t-BHP was used as positive control for oxidative stress generation which showed 6.19 and 6.48 fold increase in superoxide and secondary ROS/RNS generation.

**Figure 5:**
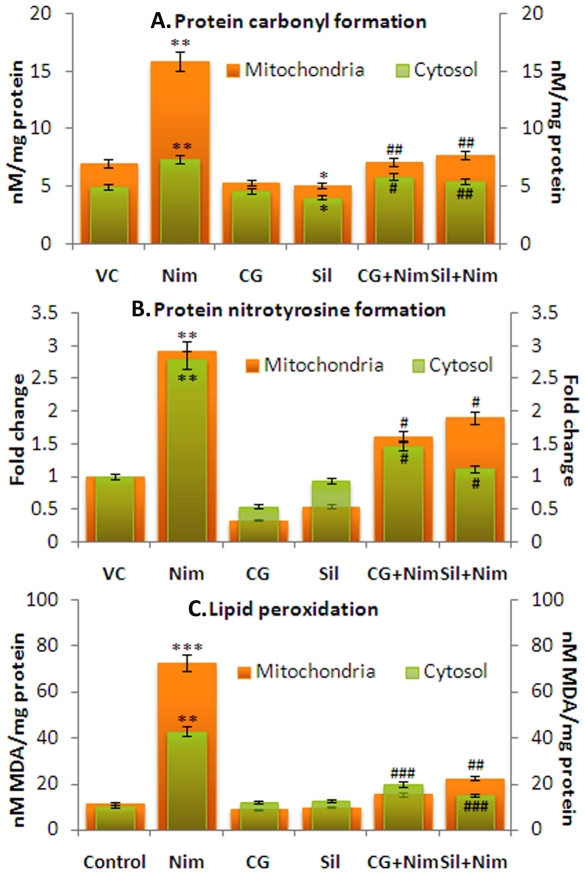
ROS/RNS induced damage to proteins and lipids. This was observed in both cytosolic and mitochondrial fractions. **A.** Protein oxidative damage is demonstrated as protein carbonyl formation (nM/mg protein), while, **B.** protein nitrosative damage as nitrotyrosine formation, and, **C.** oxidative lipid damage as MDA formation (nM/mg protein). Carbonyl and MDA formation was estimated using biochemical assays. Tyrosine formation was observed by western blot and demonstrated as fold change of densitometric values with respect to vehicle control. Level of significance is denoted as */# *P*<0.05, **/## *P*<0.01 and ***/### *P*<0.001. *, compared to vehicle control and #, compared to nimesulide stress.

In the next step oxidative/nitrosative damage to the macromolecules (levels of protein carbonyl, protein-nitrotyrosine residues and oxidative lipid damage) was observed in both mitochondria and cytosol (Figure 5A-5C). Nimesulide caused significant oxidative and nitrosative damage to the proteins of mitochondria, which was more pronounced than damage in cytosol. Nimesulide caused 1.26 and 1.93 fold (*P<0.01) increase in carbonyl and nitrotyrosine formation in mitochondria whereas in cytosol 0.47 (*P<0.05) and 1.79 fold (*P<0.01) increase was observed. CG pre-administration significantly prevented this oxidative and nitrosative damage in proteins, which was found to decrease by 1.25 (^#^ *P*<0.01) and 1.32 (^#^ *P*<0.05) fold respectively in mitochondria, and 0.30 and 1.32 fold (^#^ *P*<0.05) in cytosol when compared to nimesulide treated group. In silymarin pre-administered rats, 1.17 (^#^ *P*<0.01) and 1.03 fold (^#^ *P*<0.05) decrease in mitochondrial protein carbonyl and nitrotyrosine formation was observed.

Nimesulide also caused lipids peroxidation (MDA formation) in mitochondria (5.41 fold; *P<0.001 fold) and cytosol (3.18 fold; *P<0.01) when compared to control, suggesting that mitochondrial lipids are more prone to oxidative damage. CG pre-administration significantly prevented oxidative damage to lipids, which was found to decrease by 5.0 (^#^ *P*<0.001) fold in mitochondria and 2.24 fold (^#^ *P*<0.01) in cytosol when compared to nimesulide treated group. In silymarin pre-administered rats, 4.43 fold (^#^ *P*<0.001) decrease in mitochondrial lipid damage was observed, whereas 2.70 fold (^#^ *P*<0.001) decrease in cytosol was observed. Data suggests that pre-administration of CG accorded more protection in mitochondria than silymarin.

Results obtained so far indicate that nimesulide has potential to modulate antioxidant status by altering gene expression and activity of enzymes. Nimesulide also caused significant thiol alteration, critical for maintaining redox balance, resulting into increased oxidative stress and macromolecular damage during the process causing hepatotoxicity which was prevented by CG.

### CG Prevented Nimesulide-induced Loss of Mitochondrial Activity

Mitochondrial activity can be checked by various techniques including measurement of electron flow, NAD(P)H (reduced pyridine nucleotides) level, important for electron transport chain or by observing mitochondrial depolarization (Ψm_Low_) which is a characteristic feature in mitochondrial dysfunction and loss of activity [2].

#### CG prevented nimesulide-induced loss of mitochondrial electron flow

**Figure 6:**
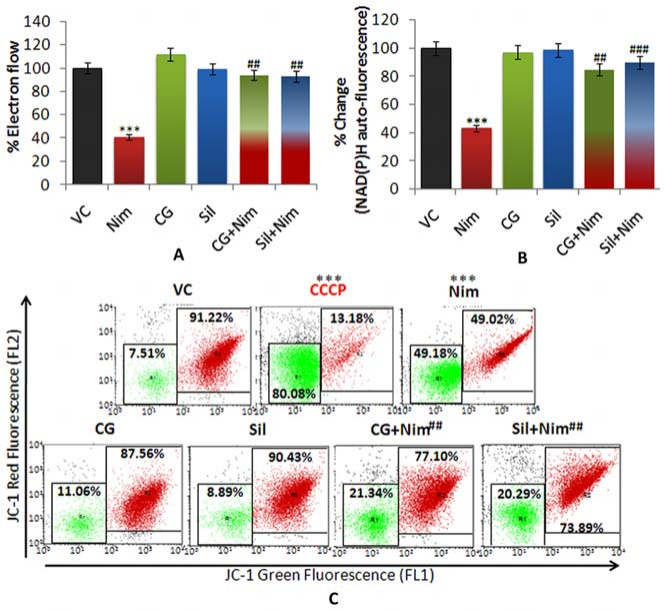
Mitochondrial Activity. A and **B.** %MTT activity as mitochondrial electron flow and UV auto-fluorescence of mitochondria as reduced pyridine nucleotides [NAD(P)H] respectively. **C.** Mitochondrial membrane potential was assessed using JC-1dye on flow cytometer. In dot plot, red fluorescence of J-aggregates represents polarized mitochondria (in right panel, red colored) whereas green fluorescence of JC-1 monomers represents depolarized mitochondria (left panel, green colored). CCCP (mitochondrial uncoupler) was used as negative control for mitochondrial membrane potential. Level of significance is denoted as **/## *P*<0.01 and ***/### *P*<0.001. *, compared to vehicle control and #, compared to nimesulide stress.

Nimesulide administration caused significant decrease (61%; *P<0.001) in mitochondrial electron flow (Figure 6A). In CG and silymarin pre-administered rats, significant prevention (^#^ *P*<0.01) in loss of electron flow was observed which was 94% (CG) and 93% (silymarin) and comparable to vehicle control group. Thus, the results indicate that pre-administration of CG prevented loss of electron flow during nimesulide stress.

#### CG prevented decrease in reduced pyridine nucleotides caused by nimesulide

In nimesulide stressed rats, a significant decrease of 57% (*P<0.001) in the level of NAD(P)H was observed when compared to control (Figure 6B). In CG and silymarin pre-administered rats significantly elevated level of NAD(P)H was found i.e. only 15% and 10% oxidation was observed and the level was comparable to mitochondria of untreated rats.

#### CG prevented nimesulide-induced mitochondrial depolarization (Ψm_Low_)

Nimesulide caused significant mitochondrial depolarization (Ψm_Low_) (49%, *P<0.001) (Figure 6C). CG and silymarin pre-administration prevented Ψm_Low_ and only 21% and 20% (^#^ *P*<0.01) mitochondria were depolarized, as compared to nimesulide treated group. Carbonyl cyanide 3-chlorophenylhydrazone (CCCP), an uncoupler, was used as positive control for Ψm_Low_ which showed 80% Ψm_Low_.

From the above data, it can be stated that nimesulide caused significant decrease in mitochondrial activity that was effectively prevented by pre-administration of CG.

### CG Prevented Nimesulide-induced Membrane Permeability Transition and Large Amplitude Swelling of Mitochondria in Vivo

**Figure 7:**
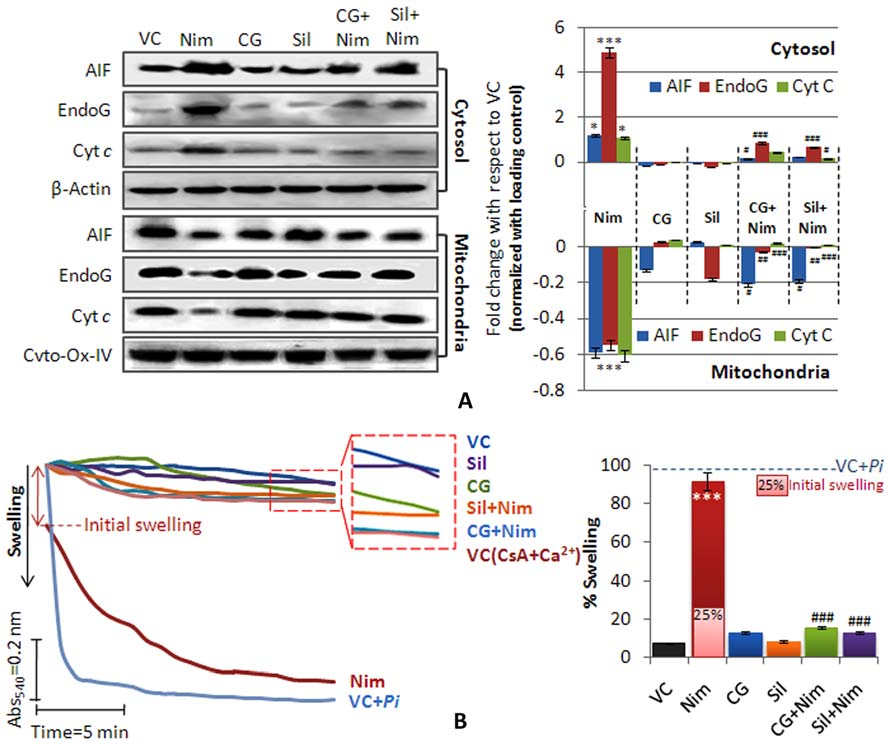
Mitochondrial membrane permeability transition (MPT). A. Immunoblot analysis of released proteins: AIF, EndoG and Cyt *c* from mitochondria to cytosol were assessed and dansitometric analysis was done. Cyto-Ox-IV and β-Actin were used as internal loading controls for mitochondrial and cytosolic proteins respectively. All the densitometric values are normalized with respective controls and values are represented as compared to vehicle control in fold change. Densitometry of bands was done using ImageJ software (V1.41o, NIH, USA). **B.** Mitochondrial swelling as a function of MPT change was also observed as decrease in absorbance at 540 nm. Cyclosporine A (CsA, MPT inhibitor) + Ca^2+^ and *Pi* (KH_2_PO_4_, MPT inducer) were used as controls of MPT. During *in vivo* treatment nimesulide showed some (25%) initial swelling after that in *ex vivo* condition during the experiment remaining swelling was observed. Dotted line in bar graph showed 100% swelling induced by *Pi.* Level of significance is denoted as */# *P*<0.05, **/## *P*<0.01 and ***/### *P*<0.001. *, compared to vehicle control and #, compared to nimesulide stress.

In the event of membrane permeability change, mitochondria specific cell death proteins, like AIF, EndoG and Cyt *c* are released from mitochondria to the cytosol. Figure 7A shows significant release of AIF, EndoG and Cyt *c* from mitochondria to cytosol during nimesulide stress in rats. In cytosolic fraction enhancement in the level of these proteins; 1.20 fold AIF (*P<0.05), 4.90 fold Endo G (*P<0.001) and 1.10 fold Cyt *c* (*P<0.05) was observed indicating translocation from mitochondria. At the same time these proteins were significantly (*P<0.001) decreased in mitochondrial fraction (AIF, 0.59; EndoG, 0.54 and Cyt *c*, 0.61 fold respectively). In CG and silymarin pre-administered rats, release of proteins was significantly (^#^ *P*<0.05 to ^#^ *P*<0.001) prevented as compared to nimesulide treated group. It was only 0.15, 0.86 and 0.43 fold increase in the levels of AIF, EndoG and Cyt *c* in cytosol whereas 2.1, 0.27 and 0.24 fold decrease in mitochondria in CG pre-administered group was observed. Silymarin pre-administered group showed similar response.

The results show that CG and silymarin when pre-administered gave significant protection to mitochondrial membrane and only non-significant alterations in the levels of AIF, EndoG and Cyt *c* were observed.

Mitochondrial swelling is an important parameter to assess membrane permeability transition. Figure 7B shows total 92% swelling of mitochondria (^*^ *P*<0.001, 25% initial swelling, before starting the kinetics; and 67% swelling during the experimental incubation) indicating compromised membrane permeability under nimesulide stress. *Pi* (KH_2_PO_4_; 2 mM) induced swelling was taken as 100% swelling. Mitochondria from CG and silymarin pre-administered rats did not show initial swelling and only 15% and 13% swelling was observed during experimental incubation of 20 min, which was non-significant and comparable to the vehicle control. Cyclosporin A (CsA; 1uM), an MPT blocker, was used as negative control and recorded only 16% swelling.

### CG Prevented Nimesulide-induced Caspase-9 and Caspase-3 Cleavage

**Figure 8:**
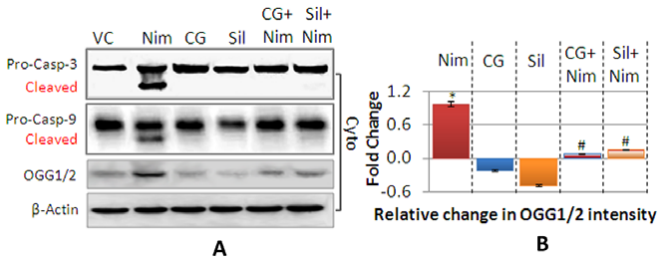
Activation of Caspase-9/caspase-3, and DNA damage. Cleavage of procaspase into cleaved low molecular weight protein as activation of caspases, was observed in cytosolic fraction and OGG formation, marker for oxidative DNA damage were assessed by immunoblotting (**A**). β-Actin was used as internal loading control and the densitometric values in bar graph (**B**) are normalized with it. Densitometry of bands was done using ImageJ software (V1.41o, NIH, USA). Values are represented as compared to vehicle control in fold change. Level of significance is denoted as */# *P*<0.05, **/## *P*<0.01 and ***/### *P*<0.001. *, compared to vehicle control and #, compared to nimesulide stress.

Release of Cyt *c* from mitochondria is an important trigger for activation of caspases. Nimesulide was found to cause cleavage of pro-caspase-9/-3 *in vivo* (Figure 8A) into active caspases. In CG and silymarin pre-administered rats cleavage of caspase-9/-3 was prevented. The data obtained in this study strongly suggests involvement of mitochondria dependent signaling cascade. It is known that during mitochondria dependent apoptosis, procaspase-9 is released from mitochondria to cytosol, auto-activates and forms apoptosome along with dATP, ApoAF-1 and Cyt *c*. Apoptosome finally cleaves and activates procaspase-3 into executioner caspase-3 which further triggers DNA damage during apoptosis [32].

### CG Prevented Nimesulide-induced DNA Damage

Level of OGG, a DNA repair enzyme and a marker for oxidative DNA damage [33], was evaluated in all the experimental groups of animals (Figure 8A). OGG level was found to be significantly high (*P<0.05) in nimesulide administered rats indicating nuclear DNA damage (Figure 8B). CG and silymarin pre-administration significantly prevented nimesulide-induced increase in OGG level and a 90% and 80% decrease in the enzyme level was observed as compared to nimesulide stressed group (^#^ *P*<0.05). CG and silymarin themselves caused non-significant decrease in OGG level in cytosol when compared to control.

## Discussion

Mitochondrial dysfunction is one of the crucial events involved in cell death by apoptosis. Mitochondrial changes may be more detrimental in the presence of abnormalities like oxidant-antioxidant imbalance. Oxidative stress makes mitochondria more susceptible to untoward effects of a drug resulting into cellular malfunctioning which is undetectable until a critical threshold is achieved [9], [34]. It is therefore, to be expected that mitochondrial impairment could be a relevant mechanism of drug-induced toxicity, particularly in liver, where most of the drugs get metabolized. Our previous study showed that nimesulide caused mitochondrial redox imbalance and MPT in isolated mitochondria [4].

In this study, our objective was to explore the role of oxidative status and its relation to mitochondrial dysfunction during nimesulide-induced hepatotoxicity in rat model along with assessment of preventive efficacy of a combination of terpenes. Nimesulide in overdose, as commonly encountered during the treatment of several pathological conditions (like ankylosis, osteoarthritis, etc.), induces severe hepatotoxicity, which decreases benefit to risk ratio for this widely accepted and popular drug [9], [12]. The mechanism underlying this hepatotoxicity is mitochondria mediated as nimesulide is found to accelerate ROS production, thereby ensuing oxidative stress [4], [7], [9]. The stress condition is all the more aggravated when GSH pool starts getting depleted. Nimesulide is also found to have potential to modulate antioxidant and redox enzymes (SOD, GPx and GR). In addition, it enhanced NOS levels, formed protein nitrotyrosine, and thus caused nitrosative stress.

GSH depletion along with NADPH oxidation and compromised GPx, GR activity, dismantled redox homeostasis, compromised antioxidant defense and enhanced oxidative stress. This enhanced ROS/RNS along with compromised antioxidants, proved deleterious for cellular macro-molecules like proteins, lipids and DNA causing significant damage to them, as evident from protein carbonyl, protein nitrotyrosine, MDA and OGG1/2 formation. Furthermore, this enhanced oxidative stress, which damaged macromolecules, results in mitochondrial dysfunction involving MPT change, impaired electron flow, NAD(P)H oxidation and Ψm_Low_. MPT has long been established as a physiological process which plays an important role in the toxic responses of various NSAIDs caused by mega pore complex formation. It induces a nonselective increase in the permeability across the mitochondrial membranes, to the compounds up to 1.5 kDa, and leads to a collapse of the membrane potential [4], [7], [9]. This process is Ca^2+^ dependent and CsA sensitive but is also regulated by a number of other factors like oxidative stress or low ATP levels [7], [35], resulting in mitochondrial uncoupling and matrix expansion (swelling). Mitochondrial swelling ultimately disrupts the outer membrane, thus releasing inter-membrane space located pro-apoptotic factors like Cyt *c,* AIF and EndoG. Cyt *c* with procaspase-9 and ApoAF-1 in the presence of dATP leads to cleavage and activation of procaspase-9 followed by caspase-3 [14], [31]. Along with AIF and EndoG translocation, caspase-3 caused significant DNA damage as evident from increased OGG level, a DNA repair enzyme, confirming involvement of mitochondria in nimesulide-induced cell death pathway. We have demonstrated that nimesulide caused collapse of the Ψm leading to impaired electron flow, which is associated with the production of superoxide. Uncoupling alone usually has a negative feedback on ROS production [31], but ROS, when generated with MPT, tends to enhance the MPT in self-propagating manner [36], [37]. It has also been proposed that in response to MPT, depletion in major antioxidants like GSH, along with other inter-membrane space proteins leak out into the cytosol and shifts mitochondrial redox balance towards a more pro-oxidant state [2], [31]. In addition to this, NAD(P)H gets oxidized and mitochondria undergo stress due to (i) lack of substrate (at complex I) for respiration and (ii) utilization in the maintenance of thiol redox status in the cell [2]. Resultant decrease in GSH levels in liver mitochondria leads to apoptosis and patho-physiology of human diseases [3], [9]. These observations regarding nimesulide-induced oxidant-antioxidant imbalance and mitochondrial dysfunction, may be a step in the direction of establishing some relation between oxidative stress at the sub-cellular level and drug induced hepatotoxicity.

Phytochemicals are being explored as promising supplements against various hepatic ailments as well as drug-induced hepatotoxicity [38]. In a previous study from our lab, common dietary phytochemicals, camphene and geraniol showed significant cyto-protective and antioxidant effect against t-BHP induced oxidative stress in murine alveolar macrophages [19]. Pre-administration of a combination of camphene and geraniol, in the present study, was found to significantly prevent alterations in redox homeostasis, subsequent mitochondrial dysfunction and release of apoptotic factors during nimesulide-induced hepatotoxicity. CG pre-administration abrogated ROS/RNS production and prevented GSH as well as NAD(P)H oxidation, in turn preventing redox-imbalance. Besides this, CG also prevented nimesulide-induced MPT, impaired electron flow, and Ψm_Low_. Even cleavage of procaspase-9 and procaspase-3 into their active form was prevented, in turn putting a brake on subsequent DNA damage and apoptosis. Thus, the capacity to promote endogenous anti-oxidative defense system and subsequently prevent important events in apoptotic cascade makes CG a promising prophylactic agent in oxidative stress related mitochondria mediated liver injury.

**Figure 9:**
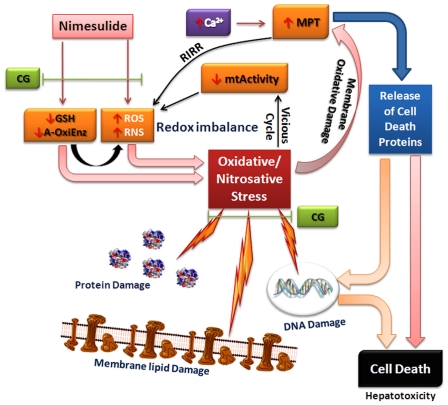
Possible role of CG administration in nimesulide-induced hepatotoxicity. In our study, nimesulide was found to enhance ROS/RNS generation and compromise antioxidant defenses in mitochondria leading to oxidative stress. As a consequence of oxidative stress mitochondrial electron flow and NAD(P)H decreased along with significant mitochondrial depolarization. Oxidative stress along with mitochondrial dysfunction facilitated membrane permeability transition (MPT). Cell death proteins like AIF, EndoG, Cyt *c* released from mitochondria to cytosol due to MPT. In such a condition, where significant oxidative stress ensued and antioxidant defenses were compromised at the transcriptional level, significant macromolecular damage occurred. Apoptotic protein Cyt *c* along with other factors like dATP, ApoAF-1 and caspase-9 activated effector caspase-3 leading to DNA damage and cell death. A combination of terpenes, camphene and geraniol (1∶1), showed their potency to prevent nimesulide-induced imbalance in oxidant-antioxidant homeostasis and its downstream effects in mitochondrial dysfunction during hepatotoxicity.

In conclusion, nimesulide caused significant decrease in cellular antioxidants and increased ROS/RNS generation which in turn caused oxidative stress. Compromised antioxidant status favored enhanced oxidative stress that facilitated depolarization of mitochondria and altered its functions (Figure 9). Dysfunction in mitochondria further increased ROS/RNS generation, which creates a vicious cycle. Enhanced oxidative stress also damaged macromolecular network, increased MPT followed by the release of mitochondrial cell death proteins, like AIF, EndoG, Cyt *c* and activated caspases (caspase-9 and caspase-3) culminating into DNA damage and cell death during hepatotoxicity. A combination of terpenes i.e. camphene and geraniol, was found to prevent oxidant-antioxidant imbalance and its downstream apoptotic events during nimesulide-induced hepatotoxicity. Therefore, the use of these natural antioxidants may improve benefit to risk ratio of nimesulide and may help to increase its use without adverse effects.
